# The Study for Diagnostic Value of β-Catenin Immunohistochemistry Marker in Distinction of Aggressive and Non-Aggressive Basal Cell Carcinoma

**DOI:** 10.30699/IJP.14.1.52

**Published:** 2018-12-27

**Authors:** Parvin Rajabi, Mitra Heydarpoor, Ahmadreza Maghsoudi, Fatemeh Mohaghegh, Maryam Dehghani Mobarakeh

**Affiliations:** 1 *Dept. of Pathology, Isfahan University of Medical Sciences, Isfahan, Iran*; 2 *Internal Medicine Dept., Isfahan University of Medical Sciences, Isfahan, Iran*; 3 *Dept of Dermatology, Medicine School, Isfahan University of Medical Sciences, Isfahan, Iran*; 4 *Resident of Pathology, Dept. of Pathology, School of Medicine, Isfahan University of Medical Sciences, Isfahan, Iran*

**Keywords:** beta-Catenin, Carcinoma, Basal Cell, Aggressive Phase, Sensitivity, Specificity

## Abstract

**Background & Objective:**

Basal cell carcinoma (BCC) is a common skin cancer arising from the basal layer of the epidermis and its appendages. They are locally invasive, aggressive, and destructive of skin and the surrounding struc- tures. *β*-Catenin is a multifunctional protein located to the intracellular side of the cytoplasmic membrane coded by the *CTNNB1 *gene, which maps to chromosome 3p22.1. It has a critical role in cell-to-cell adhesion by linking cadherins to the actin cytoskeleton and has a central role in transcriptional regulation in the Wnt signaling pathway. We evaluated the diagnostic value of the Beta catenin immunohistochemistry marker in distinction of aggressive and non-aggressive Basal cell carcinoma.

**Methods:**

This cross sectional and descriptive-analytical study was done on archived formalin fixed, paraffin embed- ded tissue blocks in pathology library of Al-Zahra hospital in Isfahan city. We used immunochemistry to determinate the role of *β*-Catenin in aggressiveness in BCC with higher rate of relapse.

**Results:**

A total of 76 samples were evaluated in two groups (aggressive &none aggressive). The mean percentage of cytoplasmic *β*-Catenin staining in aggressive group was more significant than the other group (sensitivity: 86.8% specificity: 81.6%, PPV: 81.5% and NPV: 86.1%) and the mean percentage of membranous β-Catenin staining in non- aggressive group were significant more than the aggressive group. Intensity of membranous staining in both groups significant less than normal epithelium.

**Conclusion:**

Cytoplasmic *β*-Catenin staining in aggressive BCC is more significant than non-aggressive subtypes, so this indicates that the use of *β*-Catenin IHC marker maybe helpful in the diagnosis of aggressive BCC.

## Introduction

Basal cell carcinoma (BCC) is a common skin cancer arising from the basal layer of the epidermis and its appendages. They are locally invasive, aggressive, and destructive of the skin and the surrounding structures including bone. Individuals with a history of BCC are at increased risk for subsequent lesions. Approximately 40 percent of patients who have had one BCC will develop another lesion within five years (1, 2).

BCC is the most common cancer (70% to 75% of all cancer) in humans, especially in white- skinned people, although fortunately, the mortality is low. The incidence of BCC varies in different countries and it depends to sunlight exposure (3, 4). For example, in our country Iran, BCC is the most common cancer and 15% of all cancers was BCC and it is rising each year (5). In white populations in the United States, the incidence of BCC has increased by more than 10 percent per year, and the lifetime risk of developing a BCC is 30 percent (6). The incidence in men is 30 percent higher than in women, particularly with the superficial type (7-9). The incidence of BCC increases with age; persons between the ages 55 to 75 have about a 100-fold higher incidence of BCC than those younger than 20 (10). Established risk factors include UV light exposure (Sun exposure and Thera- peutic exposure to psoralen plus ultraviolet A light (PUVA) for cutaneous disorders), chronic arsenic exposure, therapeutic radiation, immunosuppression, and the basal cell nevus syndrome BCCs manifest a keratin profile similar to that of the lower part of the hair follicle, and that is therefore, distinct from that of the adjacent epider- mal basal layer epithelia (11-14).

*β*-Catenin is a multifunctional protein in the intracellular side of the cytoplasmic membrane coded by the *CTNNB1 *gene, which is located on chromosome 3p22.1. It has a critical role in cell-to-cell adhesion by linking cadherin to the actin cytoskeleton and has a central role in transcriptional regulation in the Wnt signaling path- way (15-17).

Indeed, upon Wnt activation, *β*-catenin translocates from the membrane to the cytoplasm and nucleus, where it interacts with transcriptional activators to modulate a number of target genes associated with increased growth, invasion and cellular transformation, such as *c-MYC*2 or *cyclin D1*. There are numerous lines of evidence to implicate the importance of β-catenin deregulation and *CTNNB1 *activating mutations in carcinogenesis (18-20).

Signaling by the Wnt family of secreted glycolipo-proteins is one of the fundamental mechanisms that direct cell proliferation, cell polarity, and cell, fate determination during embryonic development and tissue homeo- stasis (21).

In the absence of Wnt, cytoplasmic β-catenin protein is constantly degraded by the action of the Axin complex, which is composed of the scaffolding protein Axin, the tumor suppressor *adenomatous polyposis coli *gene prod- uct (APC), casein kinase 1 (CK1), and glycogen synthase kinase 3 (GSK3). CK1 and GSK3 sequentially phos- phorylate the amino-terminal region of *β*-catenin, resulting in β-catenin recognition by *β*-Trcp, an E3 ubiquitin ligase subunit, and subsequent *β*-catenin ubiquitination and proteasomal degradation (22). Different studies have shown that BCCs express keratins type 5, 6 and 14 and also alpha 2 and beta 1 integrin and CD10 in a fashion that does not correlate well to tumor subtype (23, 24).

In this study, we investigated the expression of *β*-catenin in aggressive and non-aggressive BCC patients.

## Materials and Methods

This cross-sectional and descriptive-analytical study was performed on a total of 76 tissue samples of tumors that were diagnosed with basal cell carcinoma by H&E staining on light microscopy. The study was approved by the ethics committee of Isfahan University of Medical Sciences. According to the probability of re-currency, the samples were divided into two groups; aggressive and non-aggressive (38 samples in each group). The aggressive group included: superficial–micronodular-morpheaform–metatypical and infiltrative. The non-aggressive group included: nodular-nodulocystic- fibroepithelial pinkus tumor. Exclusion criteria in this study were: 1. insufficient samples in blocks, 2. history of radiotherapy for treatment of the lesion, 3. incomplete admission data, and 4. specimens from relapse locations. Formalin-fixed and paraffin-embedded BCC blocks archived in pathology lab- oratory of al Zahra hospital, Isfahan, Iran, from 2014-2015 were used. Four micron sections were prepared from tissue blocks and stained by immunohistochemistry (IHC) for beta-catenin. The used antibody was monoclonal mouse antibody, subclass IgG1, kappa, clone CCH2 (BioGenex Company, USA).

Immunohistochemistry was performed using the streptavidin–biotin complex indirect immunoperoxidase method. Sections were dewaxed, rehydrated, and incubated with 0.3% hydrogen peroxide (H2O2) in water for 30 min to block endogenous peroxidase activity. Antigen retrieval was performed by microwaving for 20 min in 0.01 mol L citrate buffer (pH 6.0) at 750 W. After rinsing in phosphate-buffered saline (PBS), blocking the

endogenous nonspecific bindings was performed by adding a blocking serum (for 30 min) and drying subsequently. The sections were then incubated with the primary antibody overnight at 40C (anti-b-catenin antibody (. After washing with PBS, the sections were incubated with secondary broad spectrum antibody (30 min), washed with PBS, and then incubated with peroxidase-streptavidin conjugate for 30 min. The sections were developed with diaminobenzidine solution (DAB) for 10 min and 0.1% H2O2 and counterstained with hematoxylin then Mounting (25).

**Figure 1:**
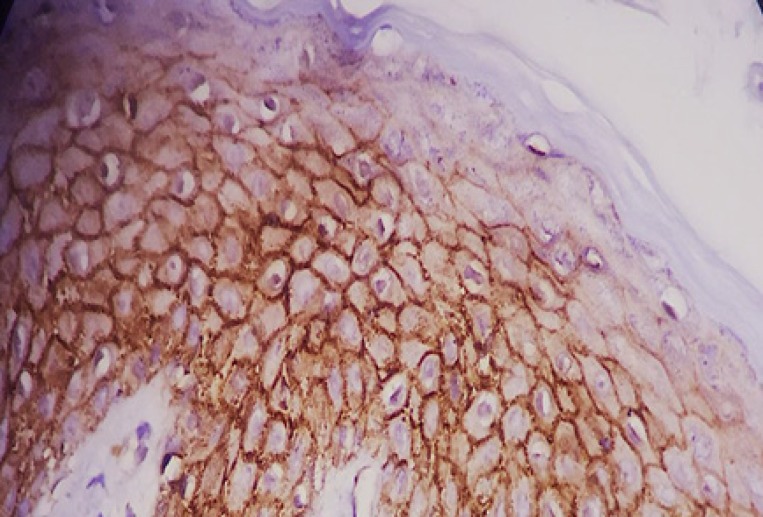
Membranous beta catenin staining in normal epithelium as positive control

**Figure 2:**
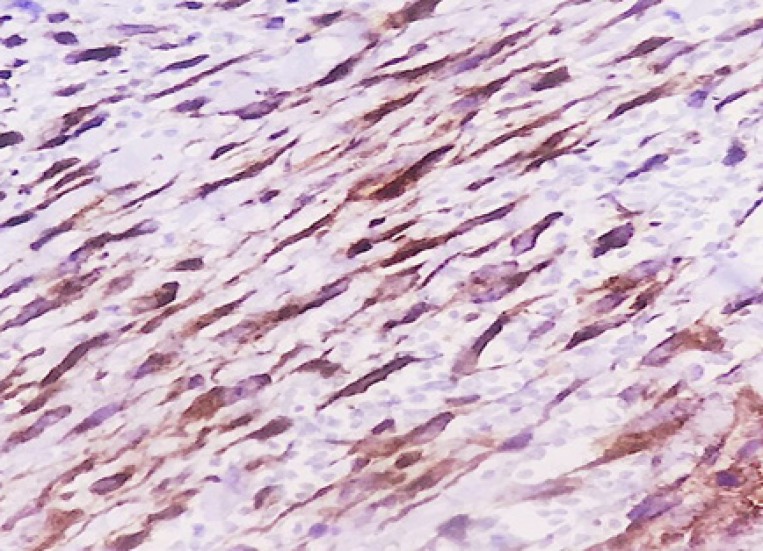
Nuclear beta catenin staining in fibromatosis as positive control

We used normal epithelium in specimens for the positive control for membranous staining. Tissue (Figure.1), samples of fibromatosis for positive nuclear staining (Figure.2), sample of endothelial cells of the vessels in lung parenchyma and pulmonary pneumocystis for the positive control for cytoplasmic staining and leiomyosarcoma for negative beta-catenin staining. We followed all patients 2 years after treatment and ask them for the relapse the tumor. Then, suspicious cases were evaluated for relapse confirmation and percentage of relapse calculated in High risk and low-risk groups. Also, the rate of accommodation was compared with data results from beta-catenin staining.

Statistical Analysis

After IHC staining and data collection, pattern (nuclear - cytoplasmic - membranous), percentage, cut off points, sensitivity, specificity, PPV and NPV of beta-catenin staining were determined and data analysis was performed in SPSS20 software and in independent T-test. Mann-Whitney, Wilcoxon rank sign Test, chi-square were used for statistical analysis.

## Results

38 samples were studied in two groups with mean age 59.11 ± 9.65 in the aggressive group and 59.53 ± 8.37 in the non-aggressive group. Statistical analysis showed no statistical difference between mean age of two groups (*P*=0.84). 47.4% of samples were female and 52.6% were male. Statistical analysis showed no statistical signifi- cant difference in the frequency of gender between groups (*P*=0.65). Also, there is no statistically significant dif- ference between the frequency of site of the tumor in groups (*P*=0.28).

**Figure 3:**
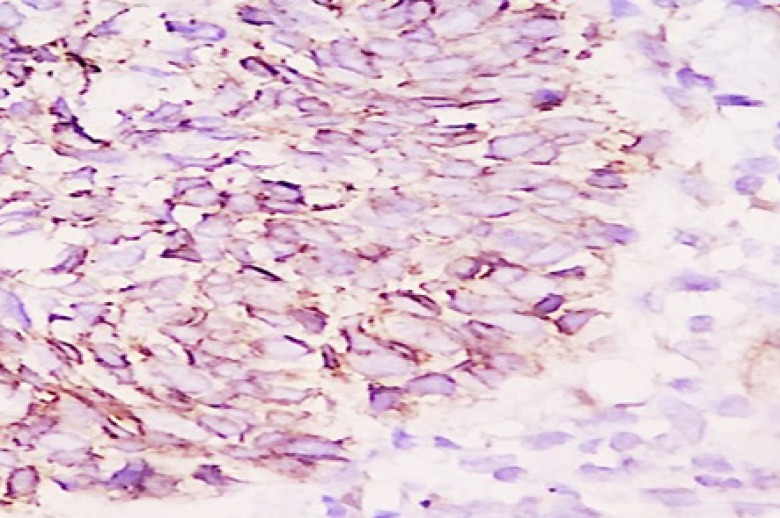
Weak membranous beta catenin staining in Non Aggressive BCC

**Figure 4:**
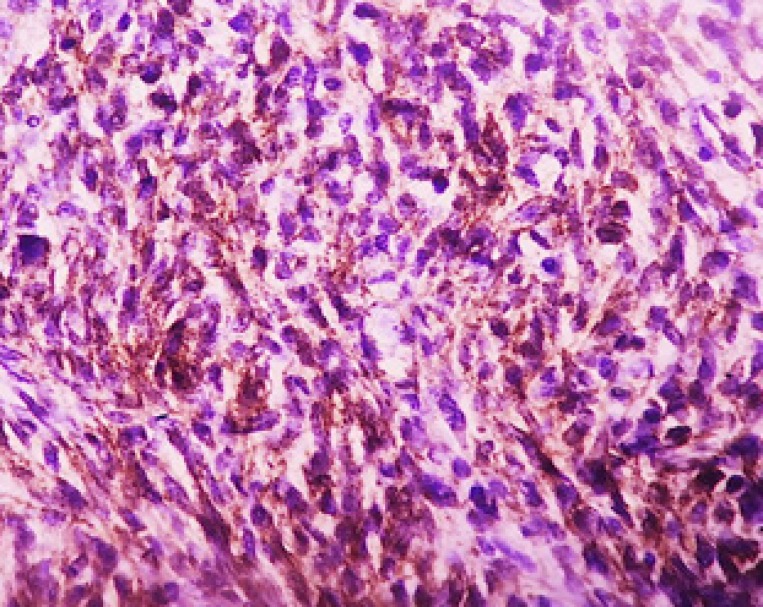
Cytoplasmic beta catenin staining in Aggressive BCC

**Figure 5:**
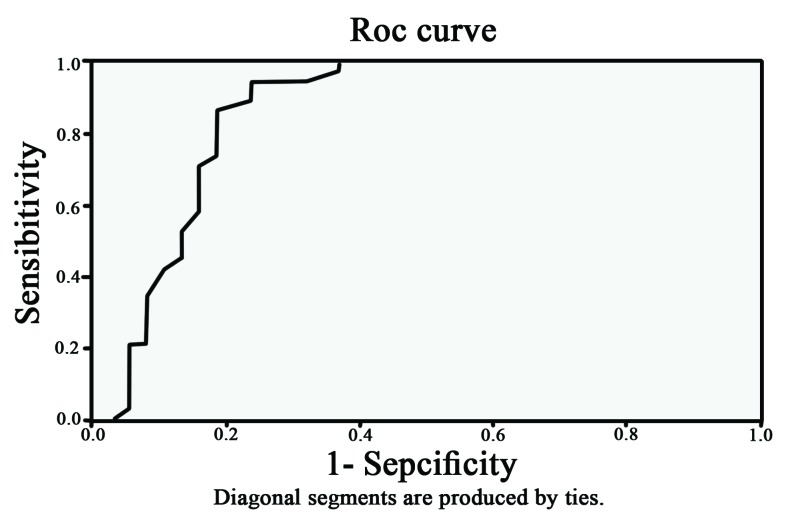
ROC curve for cytoplasmic beta catenin staining

**Figure 6:**
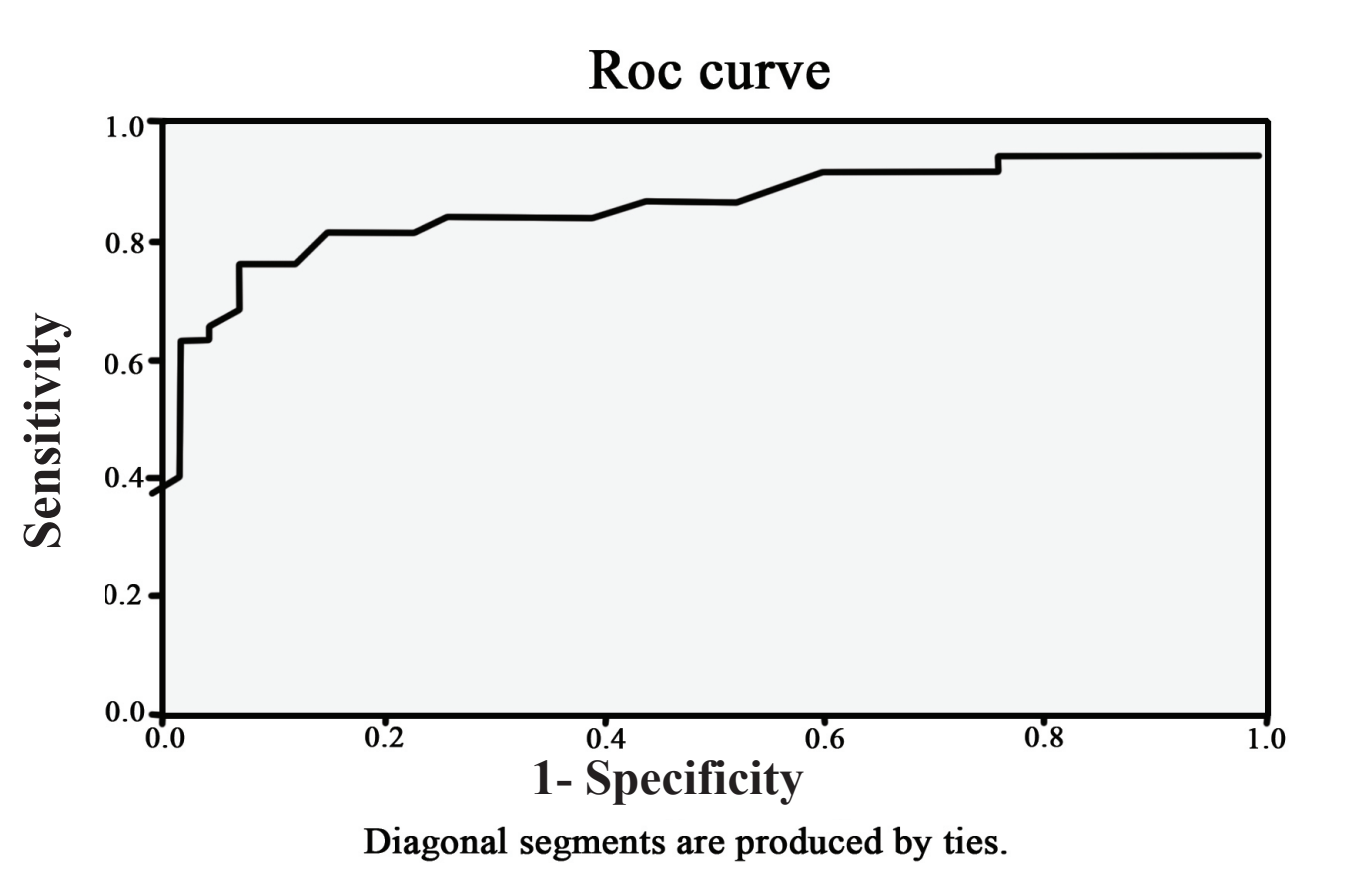
ROC curve for membranous beta catenin staining

Also we analyzed the mean percentage of cytoplasmic and membranous beta-catenin staining between the groups. Statistical analysis showed that cytoplasmic beta-catenin in the aggressive group was significantly more than in non-aggressive group (*P*˂0.001) (Figure-3). However, the mean percentage of membranous beta-catenin stain-ing in the non-aggressive group were significantly more than aggressive group (Table-1) (Figure-4) (*P*˂0.001). We used ROC curve for determining the diagnostic value in percentage for membranous and cytoplasmic beta- catenin staining. The cytoplasmic staining (Figure-5) area under the curve was 0.864 and the cut-off point was 55.5%. In this cut-off point, sensitivity: 86.8%, specificity: 81.6%, PPV: 81.5% and NPV: 86.1% were calculated. In membranous staining (Figure-6), the area under ROC curve was 0.857 and the cut-off point was 43%. In this cut-off point, sensitivity: 81.6%, specificity: 84.2%, PPV: 84.5% and NPV: 83.8% were obtained.

Also, the analysis, in both groups, showed significant intensity of membranous staining within groups was less than normal epithelium (*P*˂0.001). Moreover, our results showed that there is no statistically significant difference in the distribution of frequency of Intensity of staining in internal control between groups (*P*=0.41).

In 2 years follow up of patients, one case in each group had a relapse (2.6%), where there was no statistically significant difference between the two groups (*P*=0.25).

**Table 1:** Mean percentage of cytoplasmic and membranous beta catenin staining in aggressive and non-aggressive group

	**Aggressive**	**Non aggressive**	***P *** **value**
Variable	Mean±SD	Mean±SD	
Cytoplasmic staining	70.79±12.8	39.8±23.2	˂0.001
Membranous staining	28.8±14.0	58.8±22.6	˂0.001

## Discussion

In this study, we used 76 formalin-fixed, paraffin-embedded BCC tissue for the investigation of β-catenin expression between two groups: aggressive and non-aggressive.

Established data concerning beta-catenin IHC staining in BCC maybe an effective role in the the determination of aggressiveness in BCC subtypes. Our results showed cytoplasmic β-catenin expressed more in aggressive groups, however, membranous β-catenin was expressed more in the non-aggressive group (P value for both <0.001). Also, we demonstrated that cytoplasmic beta-catenin staining in high risk subtypes of BCC was correlated with the aggressiveness of the tumor.

Several studies demonstrated similar results to the current study. Auepemkiate et al. from Thailand (26), reported results similar to those of the current study in terms of cytoplasmic beta-catenin staining in infiltra- tive BCC and the absence of nuclear staining in another subtype. El-Bahrawy et al (25), showed that obvious decrement or loss of membranous staining with an increment of cytoplasmic staining was seen in infiltrative and morpheaform BCC, which matches with the data in this study. However, nuclear staining in infiltrative and morpheaform BCC is opposite to the results of this paper. In their study, Papanikolaou et al (27) reported that cytoplasmic and nuclear staining of beta-catenin correlate with the depth and invasion of the tumor and cytoplasmic staining in infiltrative types most seen; such data on cytoplasmic staining is the same as our study. Disaffiliation between pattern of beta-catenin staining in subtypes of BCC was reported in Saldanha et al. study, (28) as they showed that nuclear staining witnessed an increase in cell proliferation, which does not match our obtained results.

In South Korea, OH-St et al (29) reported an increment of nuclear staining in infiltrative and morpheaform subtypes and nucleocytoplasmic staining in micronodular and basosquamous BCC. Their results on cytoplas- mic staining in the aggressive group also matches the results of our study. . Shaban and et al (30) achieved the concept that nuclear expression of beta-catenin has correlated with proliferation and tumor invasion. As for loss of nuclear staining in our study, this is incompatible with our data. According to 2 years follow up of patients, around 50% of relapse in BCC happening after 2 years during the resection of primary tumor (31), an increased period of the follow up may be helpful.

## Conclusion

We studied the diagnostic value of beta-catenin IHC marker to the distinction of aggressive from non-aggres- sive BCC. Our study showed that cytoplasmic beta-catenin staining in aggressive subtypes of BCC is more significant than in non-aggressive subtypes.

## References

[1] American Cancer Society (2001). Cancer facts and figures 2000.

[2] Auepemkiate S, Thongsuksai P, Treerat P, Sirimujalin R (2005). Beta-catenin expression in relation to the histological pattern of basal cell carcinoma. Songkla Med J.

[3] Bigelow RL, Jen EY, Delehedde M, Chari NS, McDonnell TJ (2005). Sonic hedgehog induces epidermal growth factor dependent matrix infiltration in HaCaT keratinocytes. Journal of investigative dermatology.

[4] Chuang TY, Popescu A, Su WP, Chute CG (1990). Basal cell carcinoma A population-based incidence study in Rochester, Minnesota. J Am Acad Dermatol.

[5] Cui J, Zhou X, Liu Y, Tang Z, Romeih M (2003). Wnt signaling in hepatocellular carcinoma: analysis of mutation and ex- pression of beta‐catenin, T‐cell factor‐4 and glycogen synthase kinase 3‐beta genes. Journal of gastroenterology and hepatology.

[6] Deng J, Miller SA, Wang HY, Xia W, Wen Y, Zhou BP (2002). β-catenin interacts with and inhibits NF-κB in human colon and breast cancer. Cancer cell.

[7] El‐Bahrawy M, El‐Masry N, Alison M, Poulsom R, Fallowfield M (2003). Expression of β‐catenin in basal cell carcinoma. British Journal of Dermatology.

[8] Elder DE, Elanitsas R, LEVER’S Histopathology of the Skin Lippincott Williams & Wilkins.

[9] Green A, Battistutta D, Hart V (1997). Skin cancer in a subtropical Australian population: Incidence and lack of association with occupation. Occupational Health and Industrial Medicine.

[10] Green A, Williams G, Neale R, Hart V, Leslie D, Parsons P (1999 ). Daily sunscreen application and betacarotene supplementation in prevention of basal-cell and squamous-cell carcinomas of the skin: a randomised controlled trial. The Lancet.

[11] Gudbjartsson DF, Sulem P, Stacey SN, Goldstein AM, Rafnar T, Sigurgeirsson B (2008). ASIP and TYR pigmentation variants associate with cutaneous melanoma and basal cell carcinoma. Nature genetics.

[12] Hannuksela-Svahn A, Pukkala E, Karvonen J (1999). Basal cell skin carcinoma and other nonmelanoma skin cancers in Finland from 1956 through 1995. Arch Dermatol.

[13] Hatsell S, Rowlands T, Hiremath M (2003). Beta-catenin and Tcfs in mammary development and cancer. J Mammary Gland Biol Neoplasia.

[14] He TC, Sparks AB, Rago C, Hermeking H, Zawel L, Da Costa LT (1998). Identification of c-MYC as a target of the APC pathway. Science.

[15] He X, Semenov M, Tamai K, Zeng X (2004). LDL receptor-related proteins 5 and 6 in Wnt/beta-catenin signaling: arrows point the way. Development.

[16] Karagas MR, Stukel TA, Greenberg ER, Baron JA, Mott LA, Stern RS (1992). Risk of subsequent basal cell carcinoma and squamous cell carcinoma of the skin among patients with prior skin cancer. Jama.

[17] Lin SY, Xia W, Wang JC, Kwong KY, Spohn B, Wen Y (2000). β-catenin, a novel prognostic marker for breast can- cer: its roles in cyclin D1 expression and cancer progression. Proceedings of the National Academy of Sciences.

[18] Logan CY, Nusse R (2004). The Wnt signaling pathway in development and disease. Annu Rev Cell Dev Biol.

[19] Nagasawa Y, Miyoshi Y, Iwao K (1999). Transformation and morphological changes of murine L cells by transfection with a mutated form of beta-catenin. Cancer Res.

[20] Naylor MF, Boyd A, Smith DW, Cameron GS, Hubbard D, Neldner KH (1995). High sun protection factor sunscreens in the suppression of actinic neoplasia. Archives of dermatology.

[21] Oh ST, Kim HS, Yoo NJ, Lee WS, Cho BK, Reichrath J (2011). Increased immunoreactivity of membrane type‐1 matrix metalloproteinase (MT1‐MMP) and β‐catenin in high‐risk basal cell carcinoma. British Journal of Dermatology.

[22] Papanikolaou S, Bravou V, Gyftopoulos K, Nakas D, Repanti M, Papadaki H (2010). ILK expression in human basal cell carcinoma correlates with epithelial–mesenchymal transition markers and tumour invasion. Histopathology.

[23] Pentel M, Helm KF, Maloney MM (1995). Cell surface molecules in basal cell carcinomas. Dermatol Surg.

[24] Plumb SJ, Argenyi ZB, Stone MS, De Young BR (2004). Cytokeratin 5/6 immunostaining in cutaneous adnexal neoplasms and metastaticadenocarcinoma. TheAmerican journal of dermatopathology.

[25] Robinson JK (1987). Risk of developing another basal cell carcinoma A 5-year prospective study. Cancer.

[26] Saldanha G, Ghura V, Potter L, Fletcher A (2004). Nuclearb-catenin in basal cell carcinoma correlates with increased pro- liferation. British Journal of Dermatology.

[27] Scotto J, Fears TR, Fraumeni JF Incidence of nonmelanoma skin cancer in the United States.

[28] Shaban M, El Masry E (2011). β-catenin in pathogenesis of basal and squamous cell Role of carcinomas: an immunohisto- chemical study. Egyptian Journal of Pathology.

[29] Shimizu N, Ito M, Tazawa T, Sato Y (1989). Immunohistochemical study on keratin expression in certain cutaneous epithelial neoplasms Basal cell carcinoma pilomatricoma and seborrheic keratosis. The American journal of dermatopathol- ogy.

[30] Sørensen HT, Mellemkjær L, Nielsen GL, Baron JA, Olsen JH, Karagas MR (2004 ). Skin cancers and non-hodgkin lym- phoma among users of systemic glucocorticoids: a population-based cohort study. Journal of the National Cancer Institute.

[31] Sparks AB, Morin PJ, Vogelstein B, Kinzler KW (1998). Mutational analysis of the APC/β-catenin/Tcf pathway in colorectal cancer. Cancer research.

[32] Stacey SN, Gudbjartsson DF, Sulem P, Bergthorsson JT, Kumar R, Thorleifsson G, Sigurdsson A, Jakobsdottir M, Sigurgeirsson B, Benediktsdottir KR, Thorisdottir K (2008). Common variants on 1p36 and 1q42 are associated with cutane- ous basal cell carcinoma but not with melanoma or pigmentation traits. Nature genetics.

[33] Stern RS, Liebman EJ, Väkevä L (1998). Oral psoralen and ultraviolet-A light (PUVA) treatment of psoriasis and persistent risk of nonmelanoma skin cancer. JNCI: Journal of the National Cancer Institute.

[34] Telfer NR, Colver GB, Morton CA (2008). Guidlines for the management of basal cell carcinoma. British journal of Derma- tology.

[35] Tetsu O, McCormick F (1999). Beta-catenin regulates expression of cyclin D1 in colon carcinoma cells. Nature.

[36] Yada K, Kashima K, Daa T, Kitano S, Fujiwara S, Yokoyama S (2004). Expression of CD10 in basal cell carcinoma. The American journal of dermatopathology.

